# Combining information from genome-wide association and multi-tissue gene expression studies to elucidate factors underlying genetic variation for residual feed intake in Australian Angus cattle

**DOI:** 10.1186/s12864-019-6270-4

**Published:** 2019-12-06

**Authors:** Sara de las Heras-Saldana, Samuel A. Clark, Naomi Duijvesteijn, Cedric Gondro, Julius H. J. van der Werf, Yizhou Chen

**Affiliations:** 1 School of Environmental and Rural Science, University of New England, Armidale, NSW Australia; 2 Department of Animal Science, Michigan State University, East Lansing, MI USA; 3 Department of Primary Industries, Elizabeth Macarthur Agricultural Institute, Menangle, NSW Australia

**Keywords:** Residual feed intake, GWAS, QTL, RNA-seq, Gene expression, Angus, Beef cattle

## Abstract

**Background:**

Genome-wide association studies (GWAS) are extensively used to identify single nucleotide polymorphisms (SNP) underlying the genetic variation of complex traits. However, much uncertainly often still exists about the causal variants and genes at quantitative trait loci (QTL). The aim of this study was to identify QTL associated with residual feed intake (RFI) and genes in these regions whose expression is also associated with this trait. Angus cattle (2190 steers) with RFI records were genotyped and imputed to high density arrays (770 K) and used for a GWAS approach to identify QTL associated with RFI. RNA sequences from 126 Angus divergently selected for RFI were analyzed to identify the genes whose expression was significantly associated this trait with special attention to those genes residing in the QTL regions.

**Results:**

The heritability for RFI estimated for this Angus population was 0.3. In a GWAS, we identified 78 SNPs associated with RFI on six QTL (on BTA1, BTA6, BTA14, BTA17, BTA20 and BTA26). The most significant SNP was found on chromosome BTA20 (rs42662073) and explained 4% of the genetic variance. The minor allele frequencies of significant SNPs ranged from 0.05 to 0.49. All regions, except on BTA17, showed a significant dominance effect. In 1 Mb windows surrounding the six significant QTL, we found 149 genes from which *OAS2*, *STC2*, *SHOX*, *XKR4*, and *SGMS1* were the closest to the most significant QTL on BTA17, BTA20, BTA1, BTA14, and BTA26, respectively. In a 2 Mb windows around the six significant QTL, we identified 15 genes whose expression was significantly associated with RFI: BTA20) *NEURL1B* and *CPEB4*; BTA17) *RITA1*, *CCDC42B*, *OAS2*, *RPL6*, and *ERP29*; BTA26) *A1CF*, *SGMS1*, *PAPSS2*, and *PTEN*; BTA1) *MFSD1* and *RARRES1*; BTA14) *ATP6V1H* and *MRPL15*.

**Conclusions:**

Our results showed six QTL regions associated with RFI in a beef Angus population where five of these QTL contained genes that have expression associated with this trait. Therefore, here we show that integrating information from gene expression and GWAS studies can help to better understand the genetic mechanisms that determine variation in complex traits.

## Background

The incorporation of genomic information in livestock breeding programs is a common strategy to improve accuracy of selection for economically important traits. This is most useful for traits in the breeding objective that are not often measured by breeders. In beef cattle, the aim of most production systems is to select for more feed efficient animals since feed costs constitute around 70% of the total expenses [1]. The measurement of feed intake is costly, usually requiring expensive equipment to determine phenotypes for growth and feed intake in a 70 days test period in a feedlot. Feed efficiency in beef cattle is often expressed as residual feed intake (RFI) which is the difference between the observed feed intake recorded over a period of time and the expected feed intake based on the animal’s growth rate and maintenance requirement [2]. RFI reflects the variation in feed intake conditional on productivity, and therefore the variation in RFI can be used to explore the underlying causes of genetic variation using genomic technologies.

Modern genomic tools can be utilised to unravel the underlying biology of genetic variability in plants and animals. Methods that examine the heritability of traits, along with genome-wide association and gene expression studies have been utilised to attempt to understand the genetic basis underlying phenotypic differences between individuals. Numerous studies have reported variance components and heritabilities for RFI in cattle and correlations with other important production traits [3–5]. More recently, genome-wide association studies (GWAS) have been used to reveal the genomic architecture of polygenic traits by finding statistical associations between the phenotype and genetic markers assumed close to putative QTL (quantitative trait loci). Several GWAS for RFI have been performed in beef and dairy cattle [6–10]. Estimates of heritability of RFI range from low to moderate (0.14 to 0.49) and GWAS point at QTL for this trait in regions on many chromosomes (BTA3, BTA5, BTA6, BTA8, BTA12, BTA13, BTA15, BTA17, BTA18, BTA20, BTA21 and BTA22, see references 6, 7, 9). Identification of the causal variants in these QTL regions could help to better understand the genetic mechanisms underlying this trait. However, GWAS results are rarely conclusive and studies often require more phenotypic data on a larger number of animals as well as denser SNP panels to precisely locate the causative mutations, and genes involved in RFI.

Additional to GWAS, a number of studies have used transcriptomic data to find the genes that are differentially expressed with contrasting phenotypes or genotypes [11, 12], e.g. some studies have identified genes significantly associated with RFI in Angus cattle [13] and others have contrasted divergent lines or extreme phenotypes in other breeds of beef cattle [14]. However, there has been little consistency among the results of these studies. Gene expression studies are challenging, and they can vary widely in describing transcriptomic differences encompassing different tissues, breeds, sex, and age. A multi-tissue transcriptome approach combined with GWAS results may allow validation and better interpretation of GWAS findings, potentially giving a better insight into the genetic mechanisms and the biology behind this trait.

The aim of the current study was to perform a GWAS for RFI with imputed high density (770 K) genotypes in Australian Angus steers to detect significant SNPs statistically associated with phenotypic variation in RFI. Additionally, results from a multi-tissue gene expression experiment (RNA-seq) in a separate Angus population were used to further strengthen evidence for particular genes being involved in the genetic regulation of feed efficiency in beef cattle.

## Results

### Genome-wide association study

The estimated heritability for RFI based on the 2190 steers used for the GWAS was 0.3 (±0.04) using a genomic relationship matrix and after correcting for fixed effects of the contemporary groups. Genomic inflation lambda (λ) values of 0.95 show that the resulting *p*-values from the GWAS follow a chi-squared distribution and there was no sign of any systematic bias, e. g. due to population structure. From a visual evaluation (Q-Q plot), the distribution of most of the observed p-values aligned with the distribution of the expected p-values except for the significant p-values from SNPs associated with RFI (Additional file 2: Figure S1).

**Fig. 1:**
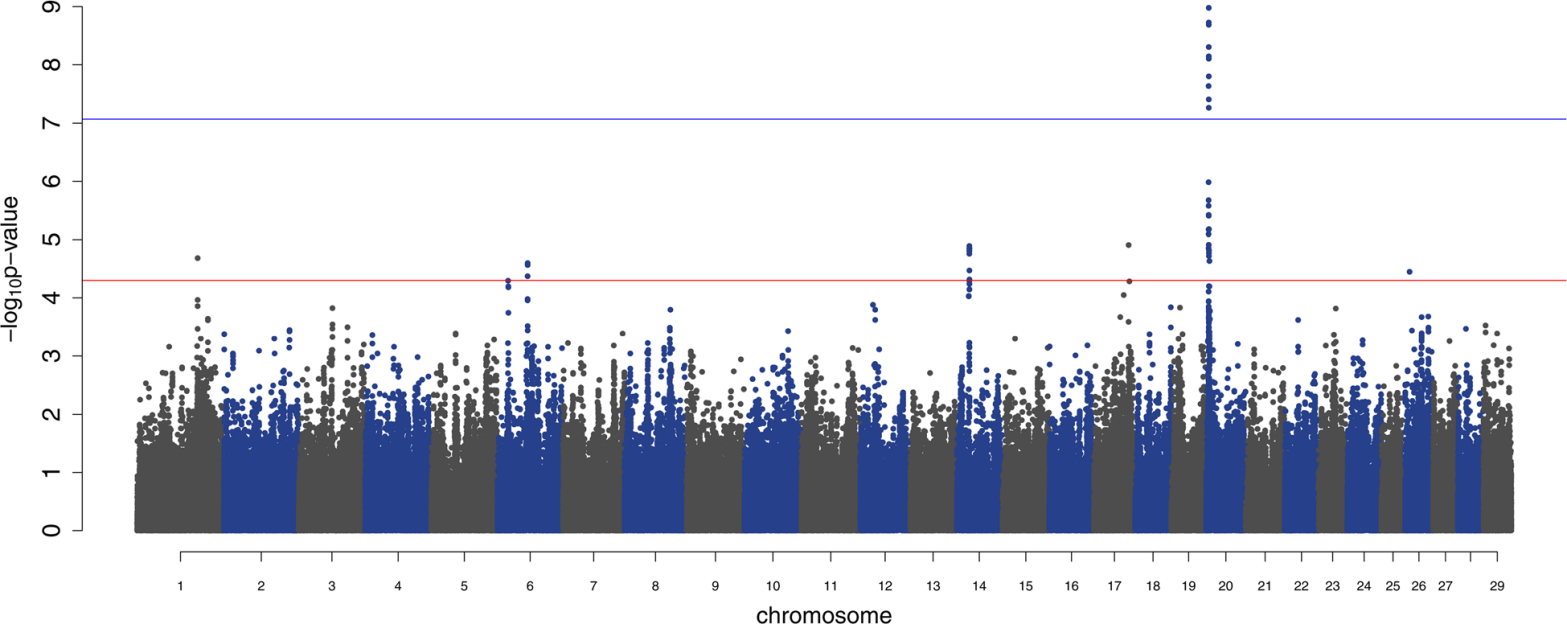
Manhattan plot of SNP’s *p*-values of association with RFI. The lines represent the significant thresholds at -log_10_(p) > 7 (blue) and -log_10_(p) > 4.3 (red)

The GWAS resulted in 78 significant SNP from six QTL regions (on BTA1, BTA6, BTA14, BTA17, BTA20 and BTA26) when using as a threshold –log_10_(p) < 5e^− 5^ (Table 1; Additional file 1: Table S1), and from these, only 11 SNPs passed the more stringent threshold (−log_10_(p) < 8.51e^− 8^), with all of these located in a single QTL on BTA20 (Fig. 1).

**Table 1:** Identified QTL associated with RFI in beef Angus (*B. Taurus* UMD3.1)

Chromosome	Regions (Mb)	Significant SNPs
1	11.05–11.06	2
6	55.18–55.08	10
14	24.18–24.39	13
17	63.63	1
20	4.88–6.12	31
26	8.91	1

Further details on all significant SNPs are shown in the Additional file 1: Table S1), while the QTL regions with the most significant SNPs can be found in Table 1. The most significant SNP overall was found on chromosome BTA20 and this SNP explained 4% of the genetic variance, while the variance explained by the most significant SNPs in each of the other QTL was smaller (< 2%) (Table 2). From the most significant SNP per region, four of them (rs42662073, rs137349090, rs42005099, rs42322957) have MAF > 0.3 while two (rs42544395 and rs133158056) have MAF < 0.09 (Table 2). The SNPs rs42005099 and rs42322957 had a negative partial dominance (d) effect of − 0.396 and − 0.336, respectively, while rs137349090 had only an additive (a) effect with animals having the AA genotype being the more efficient (i.e. the lowest phenotypic value for RFI; Additional file 3: Table S2).

**Table 2:** Association analysis of genotypes from the most significant SNP in each QTL region for RFI in Angus (*B. Taurus* UMD3.1)

SNP	Position	A1^a^	A2^b^	Fr^c^	-log_10_ (*P*)	%^d^	Effect^e^
rs42662073	20:4883142	B	A	0.45	8.98	4%	−0.26
rs137349090	17:63630684	A	B	0.34	4.91	2%	−0.19
rs42544395	14:24181858	A	B	0.08	4.89	2%	−0.34
rs42005099	1:110543274	A	B	0.49	4.68	2%	0.18
rs42322957	6:55181977	B	A	0.38	4.59	2%	0.19
rs133158056	26:8907239	A	B	0.049	4.45	2%	−0.39

^a^A1: reference allele; ^b^A2: other allele; ^c^Fr: MAF (minor allele frequency); ^d^percentage of variance explained by the genotype; ^e^SNP effect

A window of 1 Mb surrounding the most significant SNPs was used to find candidate genes with biological importance for RFI. In total, 149 genes were found in these regions and 54 among them corresponded to uncharacterized proteins (Additional file 3: Table S2). Except for the rs137349090 SNP on BTA17, which is in an intronic section of the gene *2′-5′-Oligoadenylate Synthetase 2* (*OAS2*), all other SNPs were found in intergenic regions. For the most significant SNP in this study, rs42662073 on BTA20, *Stanniocalcin 2* (*STC2*) is the closest gene. In the case of chromosomes BTA1, BTA6, BTA14, BTA26, the gene closest to the significant SNPs are *Short Stature Homeobox 2* (*SHOX2*), *LOC104968862*, *XK Related 4* (*XKR4*), and *Sphingomyelin Synthase 1* (*SGMS1*), respectively. The most relevant genes based on the biological functions reported in other studies (related to feed efficiency and growth) are summarized in Table 3.

**Table 3:** Previously reported role of the candidate genes located in the genomic regions associated with RFI in Angus

Genomic region (1 Mb)	Candidate genes	Function
BTA20 3.88–5.88	*DUSP1*-Dual Specificity Phosphatase 1	Up-regulated gene in muscle from efficient broilers [15] and adipose tissue from obese humans [16]
	*ERGIC1* - Endoplasmic Reticulum-Golgi Intermediate Compartment 1	Associated with MMWT in cattle [10]
	*RPL26L1*-Ribosomal Protein L26 Like 1	Gene associated with MMWT in cattle [10] and up regulate in breast carcinoma [17]
	STK10-Serine threonine kinase 10	Significantly associated with slaughter weight in beef cattle [18]
	*ATP6V0E1*- ATPase H+ Transporting V0 Subunit E1	Involved in oxidative phosphorylation with up-regulation in rumen epithelium of low RFI cattle [19]
	*STC2*-Stanniocalcin 2	Associated with RFI and MBW in cattle [20]; possible modulator of carcass and meat quality traits in beef cattle [21]. Overexpression resulted in postnatal growth restriction in mice [22]
	*CPEB4*- Cytoplasmic Polyadenylation Element Binding Protein 4	Gene associated with rib eye area in Nelore [23]. Nearby a suggestive SNP (*p*-value 1.38e^− 05^) for average daily gain in pig [24]
	*NEURL1B*- Neuralized E3 Ubiquitin Protein Ligase 1B	Associated with day 2 of preadipocyte differentiation in chicken [25], nearby gene to the single nucleotide variants associated with body mass index in adult humans [26], nearby gene to significant SNP for longissimus dorsi muscle area in Hanwoo cattle [27]
	*BOD1*- Biorientation Of Chromosomes in Cell Division 1	Inhibits PP2A-B56 regulating the function of Plk1 in mitotic cells at spindle poles and kinetochores [28]
BTA17 6.26–6.46	*SDS*- Serine Dehydratase, SDSL- Serine Dehydratase Like	Low expression in bovine jejunal epithelium tissue due to restricted dietary [29]
	*DTX1*- Deltex E3 Ubiquitin Ligase 1	Regulates transcription in the nucleus downstream the Notch receptor [30]
	*SLC8B1*- Solute Carrier Family 8 Member B1	Up-regulation in high-efficient broiler chickens [31]
	*OAS2*–2′-5′-Oligoadenylate Synthetase 2	Up-regulated in Blonde d’Aquitaine during embryonic muscle developmental when contrasting with Charolais [32]
	*PTPN11*- Protein Tyrosine Phosphatase, Non-Receptor Type 11	Down regulated gene in high-RFI Holstein [33], and control cell proliferation in postnatal mice [34]
	*RPL6*- Ribosomal Protein L6	Differentially expressed gene in divergent RFI lines of pigs [35]
	*LHX5*- LIM Homeobox 5	Regulates the development and distribution of Cajal-Retzius cells in the developing forebrain [36]
	*TPCN1*- Two Pore Segment Channel 1	Mice with knock down of the Tpcn1/2 had increase body mass due to faster increase in fat mass compare with the wile mice [37]
BTA14 2.31–2.51	*XKR4*- XK Related 4	SNP associated with ADFI and ADG in cattle [38], and backfat thickness in Nelore [39]
	*SOX17*- SRY-Box 17	Transcriptional regulator of differentiation in embryonic stem cells in mouse [40]. Significant SNP associated with EBV for paternal calving ease in cattle [41]
BTA1 1.10–1.11	*VEPH1*- Ventricular Zone Expressed PH Domain Containing 1	Candidate gene for rump fat thickness in Nellore [9]
	*PTX3*- Pentraxin 3	Up-regulated in breast muscle of high-feed efficient broilers [42]
	*MFSD1*-Major facilitator superfamily domain containing 1	Down-regulated gene in brainstem and hypothalamus of mice raised on high-fat diet [43]
BTA6 5.41–5.61	LOC104968862 LOC104968863 -un characterize proteins	Located in the region of SNPs for rump fat thickness [39]
BTA26 7.90–9.90	*MINPP1*- Multiple Inositol-Polyphosphate Phosphatase 1	Maintains the levels of InsP5 and InsP6 which are essential to normal cell growth [44]
	*A1CF*- APOBEC1 Complementation Factor	Splicing regulator and the A1CF loss of function elevated triglycerides levels in mice [45]
	*PAPSS2*–3′-Phosphoadenosine 5′-Phosphosulfate Synthase 2	Gene located nearby a SNP associated with DMI in feedlot steers [46]; After treating cartilage from bovine with TGF-β, the expression of gene PAPSS2 was up-regulated in articular chondrocytes, while the expression was down-regulated in cartilage from mice with negative mutation of the TGF-β receptor [47]
	*SGMS1*- Sphingomyelin Synthase 1	Gene nearby a significant SNP associated with RFI in pigs [48] and average daily feed intake [49]
	*ASAH2*- N-Acylsphingosine Amidohydrolase 2	Up-regulated in pigs with low feed conversion ratio [50]

*1Mb*: 1 M base, *BTA*: Bos Taurus Autosome

### Gene expression integration

Genes significantly associated with RFI- GSA (at *p*-value< 0.001, GSA_p < 0.001_) - in each tissue/sex were identified. The bull dataset had a higher number of GSA_p < 0.001_ with 23 (A-bulls_liver) genes in liver and 21 (D-bulls_muscle) genes in muscle, while H-steers_liver, H-heifers_blood, H-heifers_liver, and H-steers_blood datasets had 8, 6, 5, and 1 GSA_p < 0.001_ respectively (Additional file 6: Table S5). From all of the GSA, only the *Eukaryotic translation initiation factor 3H* (*EIF3H*) gene was found significantly associated with RFI, based on their expression in A-bulls_liver and D-bulls_muscle tissues. However, the expression effect was opposite in different tissues (0.101 in liver and − 0.085 in muscle).

The most significant GSA_p < 0.001_ in the QTL for RFI from all of the datasets are shown in Table 4 while the complete list is presented in the (Additional file 5: Table S4). The five most significant GSA, based on their *p*-value, were *Neuronal Regeneration Related Protein* (*NREP*), *N-Acetylated Alpha-Linked Acidic Dipeptidase Like 1* (*NAALADL1*), *Nuclear Receptor Coactivator 4* (*NCOA4*), *CD8b Molecule* (*CD8B*), and *7-Dehydrocholesterol Reductase* (*DHCR7*). On the other hand, when the GSA were ranked based on the effect that gene expression had on the phenotype, the top five were *Interferon gamma inducible protein 47* (*IFI47*), *Coiled-Coil Domain Containing 38* (*CCDC38*), *Glutathione S-Transferase Mu* 2 (*GSTM2*), uncharacterized protein (ENSBTAG00000040281), and *Retinol Binding Protein 1* (*RBP1*). The pathways related to the top significant GSA_p < 0.001_ were *Cholesterol biosynthesis*, *Fatty acid degradation*, *MAPK signaling pathway*, and *PI3K-Akt signaling pathway* (Table 4).

**Table 4:** Most significant gene expression associated with RFI and their related metabolic pathway

Symbol	Effect	*p*-value	Dataset^a^	Pathway
*NREP*-Neuronal Regeneration Related Protein	−0.21	4.66E-06	BM	MECP2 and Associated Rett Syndrome
*NAALADL1*- N-Acetylated Alpha-Linked Acidic Dipeptidase Like 1	0.33	1.33E-05	HB	–
*NCOA4*- Nuclear Receptor Coactivator 4	0.18	1.49E-05	SL	Pathways in cancer, Thyroid cancer
*CD8B*- CD8b Molecule	0.34	1.97E-05	HB	T-Cell Receptor and Co-stimulatory Signaling, Innate Immune System
*DHCR7*–7-Dehydrocholesterol Reductase	−0.29	2.32E-05	BM	Regulation of cholesterol biosynthesis by SREBP (SREBF), cholesterol biosynthesis I
ENSBTAG00000039588	0.39	3.81E-05	HB	–
*SYNE2-* Spectrin Repeat Containing Nuclear Envelope Protein 2	0.11	4.01E-05	BM	Meiosis, Ovarian Infertility Genes
*OLFML1*- Olfactomedin Like 1	−0.51	4.56E-05	BM	–
*ANGPTL2*- Angiopoietin Like 2	−0.27	5.93E-05	BM	Common Cytokine Receptor Gamma-Chain Family Signaling Pathways
*ACADSB*- Acyl-CoA Dehydrogenase Short/Branched Chain	−0.29	6.29E-05	BL	Fatty acid degradation, Valine, leucine and isoleucine degradation, Metabolic pathways, Fatty acid metabolism
*DDIT3*- DNA Damage Inducible Transcript 3	−0.22	7.26E-05	BM	MAPK signaling pathway, Protein processing in endoplasmic reticulum, Non-alcoholic fatty liver disease (NAFLD), Transcriptional misregulation in cancer
*TDRP*- Testis Development Related Protein	−0.45	1.21E-04	HL	–
*CCDC38*- Coiled-Coil Domain Containing 38	0.68	1.67E-04	BL	–
*AIM1*- Absent in melanoma 1	0.15	1.91E-04	BL	Fatty acid degradation, alpha-Linolenic acid metabolism, Metabolic pathways, Biosynthesis of secondary metabolites, Fatty acid metabolism
*PLA2G16*- Phospholipase A2 Group XVI	0.18	2.32E-04	HL	Glycerophospholipid metabolism, Ether lipid metabolism, Arachidonic acid metabolism, Linoleic acid metabolism, alpha-Linolenic acid metabolism, Metabolic pathways, Ras signaling pathway, Regulation of lipolysis in adipocytes
*TMEM135*- Transmembrane Protein 135	−0.21	2.50E-04	BM	–
*CENPM*- Centromere Protein M	0.45	2.82E-04	SL	Chromosome Maintenance, Signaling by Rho GTPases
*EIF2A*- Eukaryotic Translation Initiation Factor 2A	0.16	2.83E-04	BL	RNA transport, Protein processing in endoplasmic reticulum
*SQLE*- Squalene Epoxidas	−0.45	2.84E-04	BM	Steroid biosynthesis, Metabolic pathways, Biosynthesis of antibiotics
*GSTT3*- Glutathione S-Transferase Theta 3	0.27	2.91E-04	BL	Glutathione metabolism, Metabolism of xenobiotics by cytochrome P450, Drug metabolism - cytochrome P450, Chemical carcinogenesis
*HMGCS1*–3-Hydroxy-3-Methylglutaryl-CoA Synthase 1	−0.24	2.94E-04	BM	Synthesis and degradation of ketone bodies, Valine, leucine and isoleucine degradation, Butanoate metabolism, Terpenoid backbone biosynthesis, Metabolic pathways, Biosynthesis of antibiotics
*GHDC*- GH3 Domain Containing	0.17	3.59E-04	HL	Innate Immune System
*EIF3H*- Eukaryotic Translation Initiation Factor 3 Subunit H	0.10	3.62E-04	BL	RNA transport, Measles
*GPATCH11*- G-Patch Domain Containing 11	0.33	4.14E-04	SL	–
*NET1*- Neuroepithelial Cell Transforming 1	−0.14	4.19E-04	HL	p75 NTR receptor-mediated signaling, fMLP Pathway
*BCKDHB*- Branched Chain Keto Acid Dehydrogenase E1 Subunit Beta	0.10	4.32E-04	SL	Valine, leucine and isoleucine degradation, Metabolic pathways, Biosynthesis of antibiotics
*DNER*- Delta/Notch Like EGF Repeat Containing	−1.13	4.33E-04	BM	Signaling by NOTCH1 and NOTCH2 Activation, Transmission of Signal to the Nucleus
*ALDH5A1*- Aldehyde Dehydrogenase 5 Family Member A1	0.14	4.81E-04	BL	Alanine, aspartate and glutamate metabolism, Butanoate metabolism, Metabolic pathways
*ROBO2*- Roundabout Guidance Receptor 2	0.49	5.03E-04	BL	Axon guidance
*GSTM2*- Glutathione S-Transferase Mu 2	0.61	5.51E-04	BL	Glutathione metabolism, Metabolism of xenobiotics by cytochrome P450, Drug metabolism - cytochrome P450, Chemical carcinogenesis
*PRICKLE1*-Prickle Planar Cell Polarity Protein 1	−0.31	5.62E-04	BL	Wnt signaling pathway
ENSBTAG00000040281	0.59	6.02E-04	HB	–
*IFI47*- Interferon Gamma Inducible Protein 4	0.88	6.02E-04	SL	TNF signaling pathway
ENSBTAG00000002786	−1.10	6.42E-04	SL	–
*RBP1*- Retinol Binding Protein 1	0.55	7.01E-04	HB	Nicotinate and nicotinamide metabolism, Metabolic pathways
*UBE2D2*- Ubiquitin Conjugating Enzyme E2 D2	0.10	7.21E-04	SL	Ubiquitin mediated proteolysis, Protein processing in endoplasmic reticulum
ENSBTAG00000001489	−0.18	7.24E-04	SB	Phagosome, Gap junction
*GDPGP1*- GDP-D-Glucose Phosphorylase 1	0.32	7.40E-04	HB	–
*ITGB4*- Integrin Subunit Beta 4	0.34	8.59E-04	HL	PI3K-Akt signaling pathway, Focal adhesion, ECM-receptor interaction, Regulation of actin cytoskeleton, Hypertrophic cardiomyopathy (HCM), Arrhythmogenic right ventricular cardiomyopathy (ARVC), Dilated cardiomyopathy
*URI1*- URI1, Prefoldin Like Chaperone	0.19	9.32E-04	SL	Translational Control, Apoptosis and Autophagy

^a^*BM*: bulls_muscle, *HB*: H-heifers_blood, *SL*: H-steers_blood, *BL*: A-bulls_liver, *HL*: H-heifers_liver

**Fig. 2:**
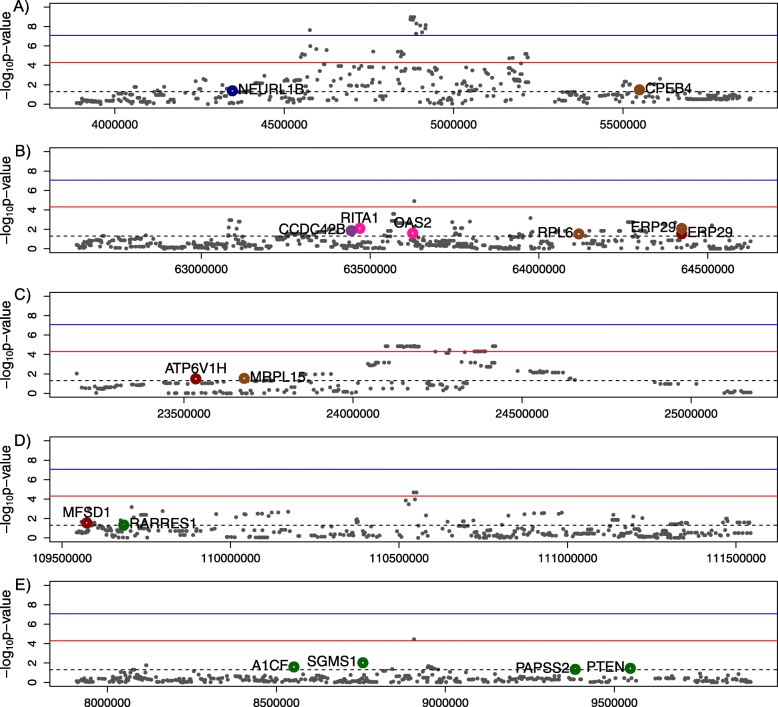
Genes significantly associated with RFI in the region of 2 MB around the significant SNPs in **a**) BTA20, **b**) BTA17, **c**) BTA14, **d**) BTA1, and **e**) BTA26. Colors indicate the corresponding dataset: bulls_liver (green), bulls_muscle (brown), steers_liver (red), steers_blood (purple), heifers_liver (blue), and heifers_blood (pink)

When the window was extended to 2 Mb, 15 genes whose expression was associated with RFI (*p* < 0.05; GSA_p < 0.05_) were identified around the top significant SNPs on BTA1, BTA14, BTA17 and BTA26. The region on BTA6 between 54 and 56 Mb does not code for any genes, therefore, there are no results for the gene expression in that region. For the most significant QTL on BTA20 positioned between 3.88 and 5.88 Mb, the GSA NEURL1B and CPEB4 were found (Fig. 2a), their expression had a positive effect on RFI (0.254 and 0.064 respectively). The region with most GSA_p < 0.05_ genes was BTA17 (Fig. 2b) with five genes (*RITA1*- *RBPJ Interacting and Tubulin Associated 1*, *CCDC42B*- *Coiled-Coil Domain Containing 42*, *OAS2*, *RPL6*- *Ribosomal Protein L6*, and *ERP29*- *Endoplasmic Reticulum Protein 29*). Gene *ERP29* was significantly associated in two datasets (H-steers_liver and D-bulls_muscle). However, similar to gene *EIF3H* mentioned before, the direction of the effect was found to be opposite in different tissues, with a regression of RFI on lcpm of 0.079 in liver and − 0.12 in muscle. The QTL region with the second most GSA_p < 0.05_ was BTA26 (between 7.90 and 9.90 Mp) with four GSA (*A1CF*- *APOBEC1 Complementation Factor*, *SGMS1*, *PAPSS2*–*3′-Phosphoadenosine 5′-Phosphosulfate Synthase 2*, *PTEN*- *Phosphatase and Tensin Homolog*) (Fig. 2e). From these genes, the gene expression of *A1CF* was down-regulated (− 0.10), while the other GSA_p < 0.05_ were up-regulated. The QTL regions with a smaller number of GSA_p < 0.05_ were BTA14 (with *ATP6V1H* and *MRPL15* between 2.31 and 2.51 Mb; Fig. 2c) and BTA1 (with *MFSD1* and *RARRES1* between 1.10 and 1.11 Mb; Fig. 2d).

## Discussion

In this study, the heritability estimated for RFI (h^2^ = 0.3) is in agreement with other estimates reported previously for other Angus populations [10, 20, 51], an Angus-Brahman herd (0.30) [52], and Nellore (0.17) [53]. However, in some other studies in Angus and Charolais populations, the heritability has been reported as high as 0.47 and 0.68, respectively [54]. Most of those studies, however, are based on relatively small data sets.

### Genome-wide association for RFI

Six QTL regions were identified to be associated with RFI on BTA1, BTA6, BTA14, BTA17, BTA20, and BTA26 (Table 1). A QTL for RFI on BTA20 has been reported in earlier studies, however, it is not the same location as in this study. The significant SNP for RFI (20_51402608) [6] was identified in Angus and is located 46.5 Mb from our most significant SNP while on chromosome 20 there was a significant QTL for ADG (BTA20_39) in SimAngus which is 34.1 Mb apart from our QTL for RFI [10]. The differences in regions found in our results compared with the regions reported in earlier studies could be due to the use of different Angus population, the number of animals used, some findings maybe false positives, or the approach applied to measure and define RFI might differ. Additionally, the fact that nearby SNPs have been previously reported as being associated for other traits (like MMWT, DMI) could be due to the pleiotropic effect of some regions. For example, the same regions have been associated for DMI-MBW, ADG-MBW, RFI-MBW [20], and RFI-DFI [6]. Although RFI and ADG and MBW had no correlation at the phenotypic level due to the conditional adjustment, there could still be a correlation at the genetic level [55], albeit relatively small. Interestingly, the gene *STC2* was the closest to the QTL on BTA20 in our study, and previous studies have reported SNPs (rs133032375) in this region significantly associated with mid-test weight and RFI in Hereford [20]. This gene *STC2* is a proteinase inhibitor of PAPP-A and the over-expression of *STC2* in mice causes a reduction in postnatal growth compared with normal mice [22, 56]. Additionally, mice with an over-expression of human *STC2* showed reducing bone and skeletal muscle growth [57].

There were five other regions identified in this study that provided further information of candidate genes with biological relevance to RFI (Table 1). On BTA1, a close QTL has been identified in BTA1_103459113 associated with RFI [6], while BTA1_106 [10], and BTA1_107 [20] were associated with feedlot dry matter intake (DMI), BTA1_108 was identified for MMWT [10]. Here we identified the nearby gene *PTX3* which previously was reported as up-regulated in breast muscle of high-efficient broilers [42]. Another gene found in the 1 Mb window from the significant SNP for BTA1 is *MFSD1* which is down-regulated in the brainstem and hypothalamus of mice raised on a high-fat diet [43].

On BTA14, the SNP rs42544395 was the most significant for RFI (Table 2), which is close to the SNP identified in SimAngus 14_17 for DMI, BTA14_24, BTA14_25 and BTA14_26 for MMWT, while BTA14_27 was associated with RFI in Angus [10]. In another population of Angus cattle, the SNP BovineHD1400006992 (BTA14_24114365) was significantly associated with PW_lwt, and SNP BovineHD1400007153 (BTA14_24621142) was associated with RFI [6]. The closest gene to SNP rs42544395 is XKR4 which was associated with feed intake and growth in cattle [38]. This gene was also reported as associated with rump fat thickness [58] and back fat [39]. In the Nellore breed, the *XKR4* gene was associated with tenderness [59].

The SNP 17_58 was earlier reported for RFI in Angus [10] and it is close to the identified QTL on BTA17 (rs137349090). Multiple interesting genes were identified in the 1 Mb region surrounding this SNP (Table 3). The *OAS2* gene seems to play an important role during muscle development [60]. Another gene, *SLC8B1*, was reported as up-regulated in high-efficient broiler chickens [31], while the gene *PTPN11* was down regulated in high-RFI Holstein [33]. Divergent RFI lines of pigs had differential expression of *RPL6* [35], another gene located close to rs137349090. The estimation of dominance effects in the most significant SNPs for each QTL showed that with the exception of SNP rs137349090, all SNPs had a significant dominance effect, with some even showing overdominance (Additional file 4: Table S3). Similarly, significant dominance and epistatic effects for carcass, growth and fertility traits were found in Angus cattle [61]. This pattern of large dominance effect is consistent with the suggestion by Jiang*,* et al. [62] that the contribution of non-additive effects to the total genetic variance for complex trait in Holstein cattle can be considerable. However, as pointed out by Hill et al. (2008), most of the dominance effects are captured by the additive genetic variance [63].

In our study, we found more negative than positive dominance effects, which is in agreement with those reported previously for RFI, age at puberty and postpartum anoestus interval [61]. The SNP rs137349090 identified on BTA17 had no dominance effects, which is a relatively accurate estimate as we found that this SNP has a sufficient number of observations for each of the genotypes (MAF = 0.34). There are two important GSA in this region (*RPL6* and *ERP29*; see Fig. 2b) that were reported as differentially expressed in divergent lines for RFI in pigs [35] and chicken [64]. Altogether, these results suggest that the information on this SNP genotype might contribute to a higher accuracy of genomic prediction of phenotype or breeding value for RFI.

Finally, we identified a QTL for RFI on BTA26, and this region has not been reported previously for RFI. In this region, the gene *SGMS1* is close to the most significant SNP rs133158056. The function of the *SGMS1* gene has not been documented, however, significant SNPs for RFI [48] and average feed intake [49] have been identified in pig for the homologous gene region. Moreover, in the present study, the expression of *SGMS1* in bulls strongly selected for RFI was significantly higher (with a positive effect 0.11) than in animals selected for low RFI.

### Gene expression overlap with GWAS results

The use of RNA-seq in multiple tissues of Angus cattle allowed us to identify the genes that were significantly associated (GSA_p < 0.05_) with RFI inside a window of 2 MB from the most significant SNPs. The genes NEURL1B and CPEB4 were located on BTA20 (Fig. 2a) and have not been reported to be associated with feed efficiency traits in previous studies, motivating further analysis of these genes to validate these results and to determine the role of these genes in relation to RFI. On BTA17, we found a relatively high number of GSA, with gene *ERP29* proximal to the SNP rs137349090. This gene has been reported previously as differentially expressed between high and low RFI lines in chickens [64]. In addition, on chromosome BTA17, the gene *RITA1* was significantly associated with RFI and is close to the significant SNP rs137349090. This gene is a tubulin-binding protein that acts as a negative regulator in the *Notch signaling pathway*. However, there is no previous report of this gene to be associated with feed efficiency traits. On BTA14, the gene *ATPase H+ Transporting V1 Subunit H* (*ATP6V1H*) was nearest to the significant SNP (rs42544395) and was significantly associated (GSA_p < 0.05_) for RFI in steer liver tissue (Fig. 2c). This gene has been reported previously for traits that define puberty (age at first corpus luteum and scrotal circumference of 26 cm) in Brahman cattle [65]. The scrotal circumference was reported to be higher in young bulls with high RFI (when backfat thickness was corrected for in the model) [66]. Nonetheless, in a previous study, there was no detrimental effect of low RFI on scrotal circumference of bulls [67]. On the same chromosome, the expression of the gene *Mitochondrial Ribosomal Protein L15* (*MRPL15*) was significantly associated (GSA_p < 0.05_) with RFI in muscle from D-bulls. Previous reports have shown low expression of *MRPL15* in double muscle *Semitendonosus* in cattle [32], but a higher gene expression was found in more feed efficient broilers [68].

We used a higher significance threshold (GSA_p < 0.001_) for a genome wide search for differentially expressed genes, and observed that the highest number of GSA_p < 0.001_ was observed in A-bulls_liver followed by D-bulls_muscle, H-steers_liver, H-heifers_blood, H-heifers_liver, and H-steers_blood with 23, 21, 8, 6, 5, and 1 GSA respectively (Additional file 6: Table S5). The higher number of GSA_p < 0.001_ in A-bulls_liver agrees with the larger variation in phenotypes for RFI found in the bulls used in this study, while the lower number of GSA in the H-groups, maybe due to the smaller variation in RFI values observed among heifer and steers in these groups (Table 6). Additionally, the small library size used in this analysis could lead to missing observations on some genes relevant in the RFI biology.

The gene *EIF3H* was found significantly associated in A-bulls_liver and D-bulls_muscle. Interestingly, this gene was observed to be over-expressed in Hanwoo cattle for animals with increasing CWT and EMA [69]. In trout, the *EIF3H* gene has been shown to be involved in compensatory muscle growth [70]. Further studies are needed to better understand the role of *EIF3H* gene on RFI in liver and muscle tissues.

The top significant genes (GSA_p < 0.001_) seem to play a role in RFI as they were shown to be associated with phenotypic differences and their functional annotation is consistent with RFI as a biological trait. The gene *NREP* for example, is close to a region associated with feed efficiency in Nellore cattle [71]. Another interesting gene is *GSTM2*, which in this study, we found positively associated with RFI in liver tissue from bulls. GTM gene family had been reported previously to be associated with RFI, *GSTM1* and *GSTM3* were highly expressed in high-RFI animals in liver [11]. *GSTM1* and *GSTM2* were significantly correlated with RFI-EBV and were up-regulated also in liver from high RFI steers [72]. The corresponding pathways in which the top significant GSA_p < 0.001_ were involved is reported in Table 4. In spite of the underpowered small library size obtained in the RNA-seq data, multiple genes could be found to have a significant effect on RFI, based on their observed expression and their potential function in pathways like *Cholesterol biosynthesis*, *Fatty acid metabolism*, *MAPK signaling pathway*, *Glycerophospholipid metabolism*, *Metabolism of xenobiotics by cytochrome P450* and *PI3K-Akt signaling pathway* (Table 4). The diversity of pathways found in our results and other studies [19] reflect many processes involved in RFI and the genetic complexity of this trait.

### Limitations of the study

This study has some limitations in the analysis of gene expression (RNA-seq). The small library size obtained from sequencing the RNA reduced the chance of finding strong GSA for RFI in the transcriptome of the animals. A pathway enrichment analysis from the obtained GSA was not significant (for the Benjamini multiple test) and was not included in this study because it could be affected by the low level of deep sequencing. However, we still consider our results meaningful and they could be used to validate results from the GWAS, overall offering more evidence and a biological interpretation of their potential role in determining genetic variation in RFI. The Angus populations used for the genomic analysis came from a commercial herd while the dataset used in the transcriptomic analysis was from animals under divergent selection lines for RFI. In addition, the animals were tested for RFI at different ages in both datasets, 18 months and 13 months for the GWAS and transcriptomic dataset, respectively. Therefore, due to these differences, results in this study, obtained by combining gene expression results in RFI contrasting phenotypes and results from genetic variants found to be associated with phenotypic variation in RFI should be interpreted with care. Nevertheless, our results encourage the use of various types of “omics” information in the same population as a way to decipher the genetic and genomic architecture of complex traits and as a way to obtain a better biological interpretation of the trait.

### Future implications

The approach followed in this study illustrated the benefit of combining genomic information with gene expression to obtain an enriched overview of the genes implicated in RFI. The genes identified in this study could be used as a target for further functional studies, to help further elucidate their role in other cattle breeds or with different diets.

In future studies, the access to whole-genome sequence and larger datasets are desired to confirm and refine the five suggestive QTL at BTA1, BTA6, BTA14, BTA17, and BTA26. The use of sequence data and larger more diverse or multi-breed population could alleviate the limitation due to linkage disequilibrium (LD), where the genotypes of multiples SNPs would be correlated with the causal variant. Furthermore, sequence information is more likely to uncover QTL in other regions. Finally, combining information from both GWAS and transcriptomic profiling could help to select the SNPs that can contribute to an increased accuracy of prediction of phenotype or breeding values for RFI.

## Conclusion

In this study, we investigated the genome-wide association of SNPs with RFI in an Australian Angus beef cattle population. We identified six QTL regions associated with RFI (BTA1, BTA6, BTA14, BTA17, BTA20 and BTA26) reflecting the polygenic nature of this trait. Promising candidate genes were identified around the most significant SNPs in each QTL. We also revealed 15 genes in these QTL regions whose expression were significantly associated with phenotypic and genetic differences in RFI (*NEURL1B*, *CPEB4*, *RITA1*, *CCDC42B*, *OAS2*, *RPL6*, *ERP29*, *ATP6V1H*, *MRPL15*, *MFSD1*, *RARRES1*, *A1CF*, *SGMS1*, *PAPSS2* and *PTEN*). Our approach demonstrates that combining GWAS and RNA-seq information improves the interpretation of GWAS results and gives it a more biological connotation.

## Methods

### Data used for the GWAS

#### Animals and phenotypes

All phenotypic data were collected on 2190 Angus steers from the Angus Sire Benchmarking Program (ASBP, also known as the Angus Beef Information Nucleus) during a feedlot testing period between 2013 and 2017. This structured dataset represented a progeny test of registered Angus sires from herds located in New South Wales and Victoria, Australia. All the procedures were managed according to the welfare guidelines established by the Australian Animal Welfare Standards and guidelines for cattle (Edition one 2013) approved by the University of New England Animal Ethics Committee (Approval No. AEC12–082). All steers, were born from fixed time AI in various herds and within each herd-year calves had a maximum variation in age of 15 days. The steers were moved from pasture feeding into a feedlot at an average of 17 months of age. Before entering the test period in the feedlot, these animals had an adjustment period of 21 days followed by a 70 d test period in the Tullimba research feedlot (30°20′S, 151°10′E, altitude 560 m) near Kingstown, NSW, Australia, as described in [73] and after the test all the animals were returned to industry owners. Daily records on feed intake and fortnightly records of body weight were used to derive average daily gain and metabolic mid-weight [1, 74] and were fitted together with age in the following model:

1 $$ {\mathrm{Y}}_i={\upbeta}_o+\upbeta 1\mathrm{AD}{\mathrm{G}}_i+\upbeta 2\mathrm{MW}{\mathrm{T}}_i+\upbeta 3{\mathrm{T}\mathrm{estGroup}}_i+{\mathrm{e}}_i $$

where Y_i_ is the daily feed intake of animal *i* (kg/day), *β*o is the regression intercept, β1 is the partial regression coefficient of feed intake on average daily gain (kg/day), β2 corresponds to the partial regression coefficient of feed intake on metabolic mid weight (kg^0.73^), β3 is the partial regression coefficient of feed intake on the feed test management group (defined as feedlot test pen within herd of origin and year), and e_i_ is the residual error in feed intake of animal *i*, therefore defined as the phenotype for residual feed intake (RFI). The mean and standard deviation for RFI are shown in Table 6, with a range in RFI from − 9.3 to 4.2 kg/day.

#### Genotypes

All 2190 steers with RFI phenotypes were genotyped using various lower density SNP panels (Table 5). Their genotypes were imputed to medium density (50 k) and then to high density (HD- 770 K) as part of an imputation performed on the wider Australian Angus population. The reference population for the 50 k imputation consisted of 11,226 animals from the Angus Australia genotyped with a number of 50 k arrays (see RefImp50k in Table 5). The reference population for the high density imputation consisted of 1069 animals, again from Angus Australia (See RefImpHD, Table 5). For each SNP chip listed in Table 5, quality control (QC) was applied where only autosomal SNPs and the SNPs with a call rate higher than a 0.6 GeneCall score were kept. Further QC was undertaken using Plink v1.90b3.42 [75], filtering out those SNPs with minor allele frequency (MAF) < 0.01, deviation from Hardy Weinberg equilibrium (*P* < 10^− 6^), and those SNPs with more than 5% missing genotypes. Only animals that had a valid genotype on more than 95% of the SNPs were kept in the analysis. The total number of animals and SNPs remaining after quality control are shown in Table 5. The 50 k reference consisted of 39.7 k SNPs after QC and the merging of 50 k reference chips. Similarly, 587,437 SNPs remained after QC for the HD reference chip. Imputation to 50 k and then HD was undertaken using FImpute v2.2 [76]. The final dataset consisted of 2190 phenotyped steers.

**Table 5:** SNPs in each chip panel (SNPs after quality control) and samples used in the imputation

SNP Chip	Number of SNPs (after QC^a^)	Number of samples
Low density panel		
GGP_8K	8753 (7569)	849 (325)
GGPLD-10 K-V2.0	9323 (7636)	980 (424)
GGPLD-20 K-V1.0	20,701 (14,062)	1189 (534)
GGPLD-20 K-V3.25	25,712 (18,786)	1138 (374)
GGPLD-26 K-V3.0	26,728 (18,187)	1344 (526)
GGPLD-30 K-V4.0	30,865 (20,560)	1556 (1)
GGPLD-9 K	8659 (6230)	574 (6)
RefImp50k		
LDMAX_SNPMap	56,955 (39,736)	3950
ZM2_SNPMap	60,911 (42,522)	778
GSTP_SNPMap	54,609 (39,706)	6673
ZOE-50 K	54,001 (37,231)	231
RefImpHD		
GGPHD-770 K	777,984 (587,437)	1069

In low density arrays only the samples indicated in parenthesis were phenotyped for RFI. ^a^*QC*: quality control

To ensure imputation accuracy was acceptable, a simple cross validation was performed. One thousand animals were extracted from the 50 k reference (RefImp50k) population, their genotypes were updated to only include the low density SNP and they were subsequently imputed back up to 50 k. The HD reference genotypes were evaluated by extracting 100 animals and keeping only the SNPs corresponding to the 50 k panel and then imputed up to HD. The accuracy of imputation was measured with the correlation between the imputed genotypes and the true genotypes. The imputation accuracy was also measured as concordance which is the proportion of SNPs with matching imputed and original genotypes. The average accuracy of imputation measured as correlation and concordance were 0.96 and 0.98, respectively, for low density imputed to 50 k; while a value of 0.99 was obtained for imputing from 50 k to high density.

### Genome-wide association study

The GWAS for RFI was performed using GCTA v1.26.0 (Yang et al. 2011) fitting the genomic relationship matrix (**G**) [77] in a univariate linear mixed model:

2 $$ {y}_{ijk}=\mu +{cg}_i+{g}_i\alpha +{a}_i+{e}_{ijk} $$

where *y* is the RFI phenotypic value, μ is the mean, *cg*_*i*_ is the effect of contemporary group i, *g*_*j*_ is a fixed effect of the allele dosage at a single SNP_j_ to contrast with fitting 3 genotypes (i.e. a covariate representing the number of “1” alleles), α is the regression coefficient for the allele substitution effect, *a*_k_ is the random additive genetic effect of animal *k*, and e_ijk_ is a random residual effect [73]. A total number of 98 contemporary groups (CG) were formed by concatenating herd of origin, year of birth, age, management group prior to the feed test, feed test management group and birth type [78]. A matrix **G** with genomic relationships among the steers was calculated based on [77] and was fitted as a covariance matrix for the additive genetic effects, i.e. var.(*a*) = ${\boldsymbol{G}\sigma}_a^2$, in order to account for residual additive genetic effects as well as for population structure effects and family relatedness. The effect of individual SNPs was estimated each time using the same estimated value for ${\sigma}_a^2$. Therefore, model [2] was run 587,437 times, once for each SNP.

To control false-positive associations, a Bonferroni correction was applied. The SNPs were considered significant when its *p*-value < 0.05/587,437 giving a threshold of -log_10_(p) > 7. Additionally, we also used a lower threshold (−log_10_(5e^− 5^) > 4.3) to identify SNPs that were not statistically significant but that could be close to genes with a biological function that could be related to RFI. QTL regions were defined as the section of the genome that contains significant SNP (−log_10_(5e^− 5^) > 4.3) extending for 1 Mb on either side of the significant SNPs.

Genomic inflation of the GWAS was calculated as the median of the chi-squared test divided by the expected median of the chi-square distribution expressed as lambda (λ). The variance explained by the significant SNP as a proportion of genetic variance was calculated as the percentage of:

$$ \frac{2{p}_i{q}_i{\alpha_i}^2}{\sigma_a^2}\ x\ 100\% $$

where *p* and *q* (=1-*p*) are the allele frequencies for the *i*-th SNP, α^2^ is the estimated additive effect of the *i*-th SNP and ${\sigma}_a^2$ is the additive genetic variance.

Candidate genes within the QTL regions were further investigated for their function. For the most significant SNPs in each QTL region, we used the model [2] but with the three genotypes for a SNP locus as a fixed class variable in a univariate linear mixed model using *MTG2* v2.09 [79] in order to estimate both additive (a) and dominance (d) effects at each SNP using the formulas [80]:

$$ a=\frac{\left(\hat{AA}-\hat{BB}\right)}{2}\kern0.24em d=\frac{\hat{AB}-\hat{AA}+\hat{BB}}{2} $$

where $\hat{AA}$ and $\hat{BB}$ are the estimated effects of the homozygous genotypes, and $\hat{AB}$ is the effect of the heterozygous genotype.

### Gene expression by RNA sequencing

#### Experimental design

The animals used for the gene expression section were an independent dataset to the animals used in the GWAS analysis. All the procedures involved in the experiment were approved by the University of New England Animal Ethics Committee (AEC 06/123, AEC14–002 and AEC14–036) and New South Wales Department of Primary Industries (NSW DPI) Animal Research Authority (ORA09/015, ORA 13/16/004).

Three cohorts of animals were used for gene expression studies. All animals used for the gene expression study came from the feed efficiency selection lines of Angus cattle at the Agricultural Research Centre, Trangie, NSW, Australia. Further details on the selection design can be found in Arthur*,* et al. [1]. The first set of animals (A-bulls) consisted of 27 young bulls born in 2005 which belonged to approximately the third generation since the start of the divergent RFI selection lines [11]. These A-bulls were selected from the highest and lowest phenotypes for RFI out of a tested cohort of 90 young bulls. All these animals were reared with their mothers on pasture, weaned at about 7 months of age, and later were reared on grazing pasture till they reached feedlot entry weight (13 months). The RFI test was conducted for 70 days in the Tullimba test station using an automated feeding system which delivers and records individual animal feed intake. Based on the performance in the RFI test, 30 animals with the lowest RFI and 30 animals with the highest RFI were chosen out of 90 for collection of liver biopsies at the end of RFI testing [11]. From those animals, only 27 animals were used in this study for the gene expression analysis. The detailed procedures of the liver biopsy were described in [11] and animals were administered by appropriate pain relief and post-operative care, as directed by the veterinary surgeon. After 2 weeks of the biopsy, the animals were returned back to Trangie Agricultural Research Centre for breeding or used for other research projects.

The second set of animals were 47 young bulls (D-bulls) which were born in 2008 and were progeny of A-bulls. The D-bulls calves were reared with their mothers on pasture till weaning (~ 230 day). After weaning, the young D-bulls were reared on grazing pasture until they reached feedlot entry weight (around 13 months). The top 25 high RFI and bottom 22 low RFI young bulls were selected to be tested for RFI at Trangie. The selection of top and bottom RFI groups were based on RFI_EBV extracted from BREEDPLAN in May 2009. Tissue biopsies were collected from the *Semitendinosus* muscle at the end of the RFI test [81].

The third cohort of 25 steers (H-steers) and 27 heifers (H-heifers) were one further generation down of the selection line and were progeny of D-bulls and born in 2012. The male calves were castrated at 4 months of age. The young male and female calves were reared with their mothers on pasture until weaning (~ 230 day). After weaning, 32 heifers and 32 steers were transferred to the NSW DPI Agricultural Research and Advisory Station (Glen Innes, NSW, Australia) and grown on native pastures until they reached feedlot entry weight of approximately 400 kg BW (~ 560 days). Before the RFI test, animals were given a 2 week period of adaptation to the feedlot ration. The RFI was measured with an automated recording system for 70 days. During this period, animals had ad libitum access to a pelleted diet which 75% grain, 10% sorghum hay, and 5% protein pellets, plus monensin, vitamins, and mineral supplement. This diet had an average energy content of 10.5 MJ metabolizable energy (ME) per kilogram dry matter and 15 to 17% crude protein. Straw was provided at an average of 0.5 kg per animal per day. The animals were transferred to a respiration chamber facility at University of New England with the same diet for RFI testing. Liver tissue was collected by biopsies at the second week after the end of RFI testing [11, 82]. In addition, peripheral venous blood samples were extracted from the tail (coccygeal) vein of cattle and it was directly placed into the PAXgene Blood RNA Tubes (Qiagen, BD, cat. no. 762165). The collection tubes were gently inverted 10 times, stored at 4 °C, and transferred to − 20 °C for long term storage.

### Phenotypic traits

The average daily gain, net feed intake, average daily feed intake and metabolic mid-test weight (MMWT) were recorded for all the 126 animals. RFI was calculated for each animal based on a linear regression model of feed intake on metabolic mid-test live weight, ADG and fitting contemporary groups as fixed effect [1]. The mean and standard deviation for each dataset used in gene expression analysis is shown in Table 6. The average RFI of the samples with RNA sequences were centred close to zero and ranged from − 1.96 to 2.62.

**Table 6:** Number of samples used in the GWAS and GSA analyses and summary statistics for RFI

Dataset (analysis)	N	Mean	SD	Min	Max
Genotyped Steers (GWAS)	2190	−2.11	1.66	−9.3	4.2
Bull liver (A-bulls: GSA)	27	0.16	0.99	−1.43	1.89
Bull muscle (D-bulls: GSA)	47	−0.02	0.96	−1.96	2.62
Heifers & Steers blood-liver (H-cohort: GSA)	52	−0.004	0.81	−1.84	1.87

*SD*: standard deviation, *Min*: minimum value, *Max*: maximum value, *GSA*: gene significantly associated, *GWAS*: genome-wide association studies

#### RNA extraction and library preparation

Total RNA was isolated from liver and muscle tissue using TRI Reagent (Ambion, Applied Biosystem, Austing, TX, USA) and PAXgene Blood RNA Kit (Qiagen BD, cat. no. 762165) was used for blood samples according to the manufacturer’s instructions. The quantity of the RNA was determined by spectrophotometer Nanodrop ND-1000 (Nanodrop Technologies) and by electrophoresis on 1% agarose gel. We assessed the integrity of the RNA on an Angilent 2100 Bioanalyzer (Agilent Technologies, CA, USA). All samples had an RNA integrity number (RIN) larger than 7. RNA samples were purified using RNeasy mini columns with DNase I (Qiagen). RNA-seq libraries were prepared using the TruSeq RNA sample preparation kit (Illumina) according to the manufacturer’s protocol. The RNA-seq libraries for A-bulls and D-bulls used the polyadenylated fraction of RNA from each animal by using modified protocol of Illumina sample preparation for RNA-Seq protocol (Illumina Inc) at AgriBio (Biosciences Research Centre, Bundoora, Victoria). The RNA-seq libraries for H-steers and H-heifers were prepared by Beijing Genomics Institute (Shengzhgen, China). RNA-seq libraries were sequenced on the HiSeq2000 sequencer (Illumina) in a 101-cycle paired end run. One hundred base paired end reads were called with CASAVA v1.8 and output in fastq format.

#### RNA-seq analysis

The software *FastQC* v0.11.5 (http://www.bioinformatics.babraham.ac.uk/projects/ fastqc/) was used to assess the quality of the RNA sequences, while *Trimmomatic* v0.36 [83] was used in the pre-processing step to remove the low-quality reads and adaptors. The software *TopHat* 2.0.5 was used with default parameters [84] mapped the cleaned reads to the bovine reference genome (*Bos taurus*, Ensembl UMD3.1) and *HTSeq* v0.6.1 [85] was used to assemble the reads. The mapping summary for all datasets is shown in (Table 7). The following steps where done in R software [86]. We filtered the genes with no expression and normalized the gene counts with the trimmed mean of M-values normalization (TMM) using the R package *edgeR* v3.18.1 [87].

**Table 7:** Alignment summary of reads to the *B. taurus* reference genome (UMD3.1 Ensembl)

Dataset	Input reads				Pair reads Mapped				Mapping rate
	Total	Average	Min	Max	Total	Average	Min	Max	
A_Liver	199,721,632	7,397,097	3,968,542	12,494,809	167,142,561	6,190,465	3,123,525	11,360,856	82%
D_Muscle	317,358,653	6,752,312	4,039,955	9,423,044	285,957,850	6,084,210	3,686,141	8,662,681	89%
H_Liver	579,132,020	10,724,667	5,173,045	11,536,836	529,820,963	10,188,865	9,397,174	10,820,527	91%
H_Blood	538,841,497	11,225,865	8,976,989	11,547,203	462,519,935	9,635,832	7,610,221	10,495,347	85%

Linear regressions for RFI on the log_2_ copies per million (lcpm) were performed separately for each dataset (A-bulls_liver, bulls_muscle, H-heifers_blood, H-steers_blood, H-heifers_liver, and H-steers_liver) to find the genes significantly associated (GSA *p* < 0.05) with RFI from the set of genes that were within a 2 Mb window from the significant QTL found in the GWAS analysis. Additionally, we used expression from all the genes in the genome to select with *p* < 0.001 GSA genes (GSA _*p* < 0.001_). Based on the sign of the regression coefficient, a positive value indicated that the gene is up-regulated showing high expression, while a negative value indicates less gene expression abundance (down-regulated) with higher values for RFI (less efficient animals).

## Supplementary information

**Additional file 1: Table S1.** Mapping complete information

**Additional file 2: Figure S1.** Q-Q plot of *p*-values for the GWAS for RFI.

**Additional file 3: Table S2.** Significant SNPs information.

**Additional file 4: Table S3.** Additive and dominance effects.

**Additional file 5: Table S4.** Information of the genes located near the significant SNPs.

**Additional file 6: Table S5.** Genes significantly associated at *p* < 0.001 with RFI.

## Data Availability

Summary data of the analysis are included in this published article as Additional files. The RNA sequences raw data are available from the National Centre for Biotechnology Information Sequence Read Archive (SRA) under the accession BioProject numbers: PRJNA393239 and PRJNA579776. The genotypes used in this study are not stored publicly, however, this data is available from the Angus Society of Australia on reasonable request.

## Abbreviations

ADG: Average Daily Gain
bp: base pairs
BP: Biological Process
BTA: *Bos taurus* autosome
CC: Cellular components
G: Genomic relationship matrix
GSA: Genes Significantly Associated
GWAS: Genome-wide Association Studies
lcpm: log_2_ copies per million
LD: Linkage Disequilibrium
MAF: Minor Allele Frequencies
Mb: Mega bases
MF: Molecular function
MWT: Metabolic mid weight
QTL: Quantitative Trait Loci
REML: Restricted maximum likelihood
RFI: Residual Feed Intake
RNA-seq: RNA sequencing
SNP: Single Nucleotide Polymorphism
TMM: Trimmed mean of M-values normalization
