# Supplementary figures and images for: Combining information from genome-wide association and multi-tissue gene expression studies to elucidate factors underlying genetic variation for residual feed intake in Australian Angus cattle
Source: BMC Genomics. 2019 Dec 6;20:939. doi: 10.1186/s12864-019-6270-4 (PMC6898931; doi:10.1186/s12864-019-6270-4)

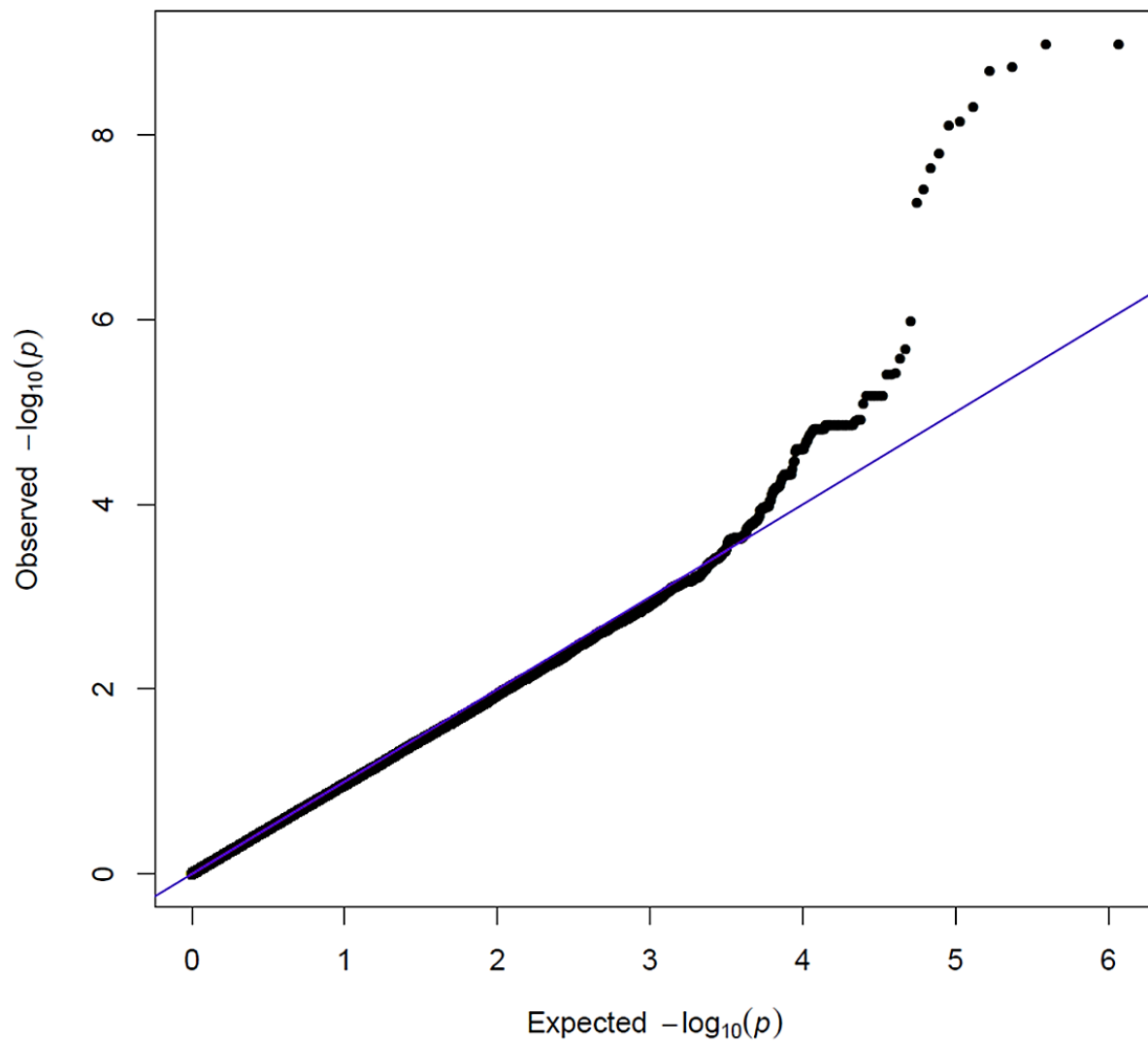

**Figure S1.** Q-Q plot of p-values for the GWAS of RFI.

Supplement: Supplementary file 2 — Additional file 2: Figure S1. Q-Q plot of p-values for the GWAS for RFI. [file 12864_2019_6270_MOESM2_ESM.pdf]
